# Improved ethanol yield and reduced Minimum Ethanol Selling Price (MESP) by modifying low severity dilute acid pretreatment with deacetylation and mechanical refining: 1) Experimental

**DOI:** 10.1186/1754-6834-5-60

**Published:** 2012-08-13

**Authors:** Xiaowen Chen, Ling Tao, Joseph Shekiro, Ali Mohaghaghi, Steve Decker, Wei Wang, Holly Smith, Sunkyu Park, Michael E Himmel, Melvin Tucker

**Affiliations:** 1 National Bioenergy Center, National Renewable Energy Lab, 1617 Cole Blvd, Golden, CO, 80127, USA; 2 Bioscience Center, National Renewable Energy Lab, 1617 Cole Blvd, Golden, CO, 80127, USA; 3 Department of Forest Biomaterials, North Carolina State University, 2820 Faucette Drive, Raleigh, NC, 27695, USA

**Keywords:** Bioethanol, Pretreatment, Enzymatic hydrolysis, Fermentation, Deacetylation, Mechanical refining, PFI mill

## Abstract

**Background:**

Historically, acid pretreatment technology for the production of bio-ethanol from corn stover has required severe conditions to overcome biomass recalcitrance. However, the high usage of acid and steam at severe pretreatment conditions hinders the economic feasibility of the ethanol production from biomass. In addition, the amount of acetate and furfural produced during harsh pretreatment is in the range that strongly inhibits cell growth and impedes ethanol fermentation. The current work addresses these issues through pretreatment with lower acid concentrations and temperatures incorporated with deacetylation and mechanical refining.

**Results:**

The results showed that deacetylation with 0.1 M NaOH before acid pretreatment improved the monomeric xylose yield in pretreatment by up to 20% while keeping the furfural yield under 2%. Deacetylation also improved the glucose yield by 10% and the xylose yield by 20% during low solids enzymatic hydrolysis. Mechanical refining using a PFI mill further improved sugar yields during both low- and high-solids enzymatic hydrolysis. Mechanical refining also allowed enzyme loadings to be reduced while maintaining high yields. Deacetylation and mechanical refining are shown to assist in achieving 90% cellulose yield in high-solids (20%) enzymatic hydrolysis. When fermentations were performed under pH control to evaluate the effect of deacetylation and mechanical refining on the ethanol yields, glucose and xylose utilizations over 90% and ethanol yields over 90% were achieved. Overall ethanol yields were calculated based on experimental results for the base case and modified cases. One modified case that integrated deacetylation, mechanical refining, and washing was estimated to produce 88 gallons of ethanol per ton of biomass.

**Conclusion:**

The current work developed a novel bio-ethanol process that features pretreatment with lower acid concentrations and temperatures incorporated with deacetylation and mechanical refining. The new process shows improved overall ethanol yields compared to traditional dilute acid pretreatment. The experimental results from this work support the techno-economic analysis and calculation of Minimum Ethanol Selling Price (MESP) detailed in our companion paper.

## Background

The United States consumes almost 19 million barrels of crude oil per day, of which approximately 58% is supplied by imports [1]. The huge demand for imported oil not only increases the market price, but it is also detrimental to the U.S. economy. The Energy Information Administration (EIA) predicts that imported oil will account for 62.5% of the total domestic oil supply by 2030 [2]. To alleviate the oil trade deficit and achieve energy independence, the production of ethanol and other transportation fuels from lignocellulosic biomass is attracting significant national interest. The National Renewable Energy Laboratory (NREL) is developing a cost-competitive bio-ethanol process using corn stover, dilute acid pretreatment and biological conversion. As disclosed in a recent techno-economic analysis report, NREL is targeting a minimum ethanol selling price (MESP) of $2.15 for this process [3]. To meet this target, much effort has been dedicated to the optimization of this process by way of parameter-selection engineering, as well as process modification.

In the NREL techno-economic analysis report, dilute acid pretreatment is performed using the conditions shown in Table 1[3]. Additional acid is added in a downstream oligomer hold reactor to convert the residual xylo-oligomers to xylose monomers at a temperature of 130°C. This process was found to convert 79.6% of the xylan to soluble xylose monomers with a 6.4% loss to furfural. Approximately 9.0% of the soluble oligomeric sugars were unreacted, and approximately 5% of the dry weight of the solids was unreacted xylan left in the solids [4]. The enzymatic hydrolysis step that follows converted an additional 82% of the unreacted xylan in the solids to xylose. Overall, an 85.3% yield of monomeric xylose was achieved from all thermochemical and enzymatic processes from experiments reported in 2010 [4].

**Table 1:** **Pretreatment conditions for corn stover in the 2011 techno-economic analysis report**[4]

**Sulfuric acid loading**	**22 mg/g dry biomass**
Residence time	5 minutes
Temperature	158°C
Pressure	5.5 atm
Total solids loading	30 wt%

However, pretreatment at such conditions brings the following problems:

● The side products and degradation products such as acetic acid and furfural produced from such pretreatment strongly inhibit the microbial growth and the final yield of ethanol [5].

● The pretreatment reactor must be constructed of exotic metal alloys to resist corrosion at the high acid concentrations and temperatures. The estimated cost of the Incoloy 825 cladded reactor for a 2,000 ton per day capacity is about $29.9 million in direct capital costs [3].

● Pretreatment at such conditions requires precise residence time control. Thus, an expensive horizontal screw pretreatment reactor is required for this process.

● The pretreatment process has high chemical costs due to the usage of relatively high concentrations of sulfuric acid, which subsequently needs neutralization before enzymatic hydrolysis and fermentation, resulting in increased usage of neutralizing chemicals. Ammonium hydroxide is used in this stage because it does not introduce salts that inhibit downstream fermentation.

● The resultant ammonium salts from neutralization require remediation by wastewater treatment. Removal of the ammonium salts requires additional caustic chemicals and aerobic wastewater digestion to convert the ammonium to sodium nitrate [3].

To overcome these problems and further lower the MESP of cellulosic ethanol, low-severity dilute acid pretreatment processes have been investigated. Studies have shown that low-severity dilute acid pretreatment converts much less xylan to degradation products, which translates to 1) lower concentrations of fermentation inhibiting compounds; and 2) more xylan available for conversion to xylose in enzymatic hydrolysis. Lower-severity pretreatments also have broader residence time control requirements and may be able to utilize lower-cost vertical reactor configurations [3]. Also, according to some vendors, lower acid loadings may allow the pretreatment reactor to be constructed using a lower-cost duplex stainless steel alloy.

However, low-severity dilute acid pretreatments also achieve low xylose monomer yields during pretreatment; rather, xylan is converted to higher percentages of non-fermentable oligomeric xylose. Low pretreatment severity also results in lower cellulose digestibility during enzymatic hydrolysis, which eventually increases the MESP. To increase the overall ethanol yield and lower the MESP, deacetylation and mechanical refining have been introduced and investigated in the current research to overcome the limitations of lower-severity dilute acid pretreatments.

Deacetylation is a unit operation designed to selectively remove acetyl groups from the hemicellulosic fraction of the feedstock prior to pretreatment [6,7]. It decreases the concentration of acetic acid released during pretreatment, which minimizes the inhibition effect of acetate salts on the downstream fermentation. Historically, acetate has been removed after pretreatment using ion exchange technologies; however, this has proven to be quite costly [8]. With the application of an alkaline extraction stage, separation of acetic acid from deacetylated feedstock may require only a supplemental dewatering or filtration step. In addition, recent studies have found that deacetylation not only significantly improves the downstream fermentability of the sugar hydrolysates but also increases sugar yields in pretreatment and enzymatic hydrolysis [6,8]. Therefore, deacetylation of the feedstock before low-severity pretreatment by alkaline extraction could potentially improve overall ethanol yield compared to standard high severity pretreatment techniques.

Mechanical refining has been widely applied in the pulp and paper industry to improve the bonding ability of the fiber and increase paper strength. It has also been used to generate microfibrils, shorten long fibers, and develop the fiber’s absorbency and porosity [9]. Disc refining has also been applied by Zhu et al. after the sulfite pretreatment of biomass to improve sugar yield during enzymatic hydrolysis [10]. Mild effects on saccharification by disc refining have been reported [10]. However, a recent study by Koo et al. reported that by applying PFI refining technology to green liquor-treated hardwood, enzymatic conversion was significantly improved, while enzyme usage was reduced by up to 50% [11]. The contradictory results are possibly due to: 1) the variation in pretreatment type and conditions, which result in different substrates; 2) the mechanical refining mechanism, because different types of refiners have been utilized; and 3) the degree of mechanical refining. Investigation of these factors has been conducted in NREL and will be reported in the future. In the current study, mechanical refining in a PFI mill has been applied to further improve the digestibility of deacetylated and lower-severity-pretreated corn stover. The PFI mill, although a laboratory mechanical refiner, provides accurate control and induces homogenous refining effects on substrate. A PFI mill is essentially a compression unit, which, given the same energy input, produces refining effect differing significantly from an conventional disc refiner [12]. Exerting more force as compression rather than shear, results in higher internal fibrillation and lower external fibrillation and fiber shortening [12]. It is also reported that during the PFI refining of never-dried softwood pulp, internal surface area of softwood fibers increased by up to 10% [13]. Similar pore volume improvement of pulp by PFI refining has also been reported by others [14,15] Many researchers have also shown internal surface area to be one of the key factors limiting biomass digestibility [16-20]. At NREL, attempts are underway to scale up the PFI mill using a Szego mill developed by Dr. Olive Trass in Toronto University. Results of these efforts will be reported in the future. For this study, acid loading and temperature in pretreatment were reduced to 0.5% (w/w) H_2_SO_4_ and 150°C, respectively. The effect of deacetylation and mechanical refining on enzymatic hydrolysis was explored at low and high solids loadings as well as on washed and unwashed substrates. Additionally, the effect of deacetylation on fermentation was studied in pH-controlled fermentations. Finally, the ethanol yields per ton of dry biomass from different pretreatment options were compared and summarized. This paper provides experimental results supporting the techno-economic analysis (TEA) in our companion paper [21].

## Results and discussion

### Pretreatment

Table 2 summarizes the xylan mass closure of pretreatment of native control and deacetylated samples from two corn stover varieties, Kramer 34 M95 and 33B51, at 150°C, 0.5% H_2_SO_4_, and 20 min residence time. Prior to pretreatment, the initial acid impregnated and dewatered corn stover is approximately 40%. Titration of this biomass shows that the H_2_SO_4_ loading is approximately 8 mg/g of corn stover, which agrees well with our calculations (7.5 mg/g of corn stover). The results of this study have been reported previously [6]. The xylan mass balances close between 100% and 103%, indicating that sampling and analytical methodologies are reliable for this process. Monomeric xylose yields for deacetylated samples were about 10% higher than for native controls (70%-73% and 52%-62%, respectively), and approached the yields at higher temperature and acid loadings reported in the literature [22,23]. Oligomeric xylose yields decreased from 21% ~ 23% for native controls to 7% ~ 10% for deacetylated corn stover, suggesting that deacetylation can significantly improve conversion of oligomeric to monomeric xylose, as discussed previously [6,7]. Furfural yields are consistently at 2%, due to the very slow rate of degradation product formation found at this pretreatment severity. Around 17% ~ 24% of xylan remains as insoluble solids after pretreatment in all cases.

**Table 2:** **Xylan mass closure of pretreatment at 150°C, 8 mg H**_**2**_**SO**_**4**_**per g of O.D. corn stover for 20 min**

	**Corn stover type**	**Xylan conversion during steam gun pretreatment**				
		**Monomer (%)**	**Oligomer (%)**	**Xylose degraded into furfural (%)**	**Xylan in solids (%)**	**Mass closure (%)**
Control	34 M95	62	21	2	18	103
	33B51	53	23	2	24	102
Deacetylated	34 M95	73	10	2	17	102
	33B51	70	7	2	21	100

### Low-solids enzymatic hydrolysis with washed solids

**Figure 1:**
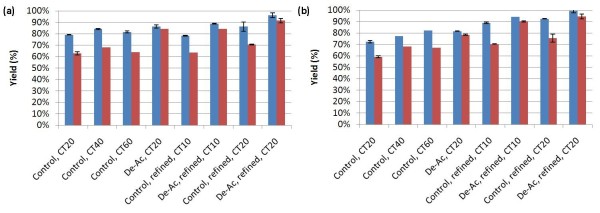
**Effect of enzyme loading, deacetylation, and mechanical refining on the digestibility of washed substrates of pretreated corn stover; (a) Kramer 34 M95, (b) Kramer 33B51; ‘blue square symbol’glucose yield; ‘red square symbol’xylose yield; (CT10: 10 mg CTec1 + 1 mg Htec1 per gram of cellulose; CT20: 20 mg CTec2 + 2 mg Htec2 per gram of cellulose; CT40: 40 mg CTec2 + 4 mg Htec2 per gram of cellulose; CT60: 60 mg CTec2 + 6 mg Htec2 per gram of cellulose).**

Low-solids enzymatic hydrolysis was performed to identify the digestibility of pretreated corn stover at near-ideal conditions. Because this assay eliminates the external mass transfer limitations that occur in high-solids enzymatic hydrolysis, the results reflect the best yields achievable for a hydrolysate. Also, the results are more easily analyzed and interpreted compared to high-solids enzymatic hydrolysis results, due to lower sampling and experimental variability. Figure 1 shows the glucose and xylose yields after 168 hrs during low-solids enzymatic hydrolysis for eight different cases. First, digestions of pretreated control stover samples with CTec2 + HTec2 were carried out at a range of enzyme loadings in order to determine whether elevated loadings would improve hydrolysate digestibility. There were minor differences in the rates (data not shown) and extents of conversion between enzyme loadings of 20 + 2 (20 mg CTec2 and 2 mg HTec2 per g cellulose; CT20), 40 + 4 (CT40), and 60 + 6 (CT60) for Kramer 34 M95. The 20 + 2 loading achieved ~80% glucan conversion, while the higher loadings achieved 80%-83% glucan conversion. The digestibility of the other corn stover variety, 33B51, lagged behind 34 M95 in both the CT20 and CT40 digestions. At the CT60 loading, it was equivalent to the others. This indicates that while biomass variability could be a significant factor in overall yields, it can be overcome with increased enzyme loadings. The convergence of conversion yields at 3X enzyme loading suggests that the issue may be one of minor differences in substrate composition or structural arrangement such that an activity not needed for one biomass variant may be limiting in another. Higher overall enzyme loadings can overcome this by either supplying more of this limiting activity or increasing the “binding pressure” that causes enzyme-substrate interaction, simply through increased enzyme loading. This effect was even more dramatic in the xylan conversion. The biomass variants gave highly varied extents of conversion at the 20 + 2 loading, ranging from ~59% to 69%. These curves began to converge at the 40 + 4 loading (68%-79%) and were even closer (64%-68%) at the 60 + 6 loading.

The controls with CT20 were also compared with deacetylated (De-Ac) samples at the same enzyme loadings. Deacetylation increased glucose yields by 7% ~ 10% and xylose yields by 18% ~ 21%. The large monomeric xylose release indicates the preference of hemicellulase enzymes for deacetylated xylan, as discussed previously in the literature [7,8]. The improved xylan removal further benefits the conversion of glucose.

As in the other digestions, pretreated corn stover exhibited increased glucan and xylan conversion after PFI refining. For control Kramer 34 M95, glucan conversion at CT10 using PFI milled pretreated corn stover (PCS) and CT20 using unrefined PCS were very similar, as minimal yield losses were observed with a 50% reduction in enzyme loading. For control Kramer 33B51, the glucose yield was improved by approximately 15% in the same case. This is very good evidence that substrate accessibility is a key limiting factor in the extent of enzymatic conversion. The size reduction resulting from the PFI refining process appears to increase the reactive surface area of the biomass, presumably exposing more substrate to the enzymes. This suggests that the slowdown and failure to release 100% of the sugars in the non-PFI-refined samples is at least partially due to a lack of enzyme accessibility to the substrate and not to a chemical barrier inherent to biomass composition. Interestingly, the xylan conversion extents for the CT10 and CT20 loadings were practically identical with refined corn stover, but much higher than with the unrefined stover. This suggests that the PFI refining has a greater effect on the digestibility of hemicellulose and that increased hemicellulose removal may be responsible for the increased digestion of the cellulose as discussed previously. The deacetylated stover varieties achieved higher conversions than the native stover did, suggesting that feedstock acetylation is an issue that must be addressed even after mechanical refining. Deacetylated PCS that was PFI-refined achieved close to 100% glucose conversion and 95% xylose conversion for both corn stover varieties, showing that carbohydrate accessibility in biomass and xylan-acetyl bond cleavage are two critical keys for the improvement of enzymatic hydrolysis yields.

### High-solids enzymatic hydrolysis with washed solids

**Figure 2:**
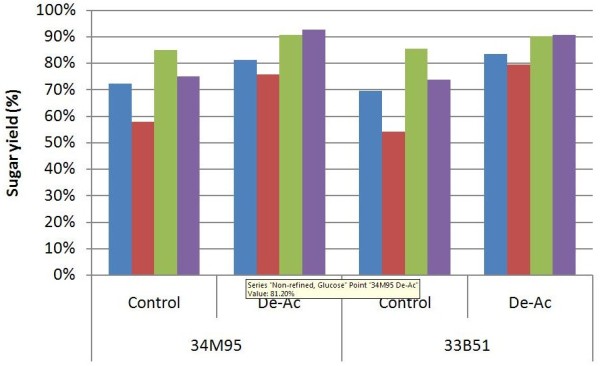
**High-solids enzymatic hydrolysis with washed solids; Enzyme is loaded as CT20; ‘blue square symbol’glucose yield, non-PFI refined; ‘red square symbol’xylose yield, non-PFI refined; ‘green square symbol’glucose yield, PFI refined; ‘purple square symbol’xylose yield, PFI refined.**

Although low-solids enzymatic hydrolysis provides an indication of the potential enzymatic digestibility of a substrate, it is not scalable to a commercial-scale process. Increasing the solids concentration during enzymatic hydrolysis and fermentation decreases the capital requirement for plant construction and reduces the energy required for product recovery [24]. However, increased solids loadings depress glucose and xylose yields due to mass transfer limitations [13]. In NREL’s 2011 design report, enzymatic hydrolysis is carried out at 20% total solids [3]. Therefore in the current study, enzymatic hydrolysis experiments are executed at 20% solids in roller bottles to provide the data required for future techno-economic analysis. Figure 2 shows the results of enzymatic hydrolysis after 120 hours with washed pretreated corn stover. The glucose and xylose yields are calculated based on the formulas developed by Zhu et al. [14].

The effects of deacetylation and mechanical refining on high-solids enzymatic hydrolysis with washed pretreated solids are very similar to the effects shown in low solids enzymatic hydrolysis; however, all yields are 5% ~ 10% lower. This is likely due to the high initial solids content limiting the flow of contained water, thus inhibiting enzyme diffusion and lowering hydrolysis yields.

Deacetylation again is shown to improve digestibility. An average yield improvement of ~10% for glucose and ~15% for xylose was achieved with the incorporation of the deacetylation process. Both deacetylated corn stover varieties showed glucose yields over 80% and xylose yields over 75%, while the yields for native corn stover samples were in the range of 69% ~ 73% (glucose) and 55% ~ 58% (xylose).

Mechanical refining also was demonstrated to improve digestibility. Samples with mechanical refining but without deacetylation achieved ~85% glucose yields and about 75% xylose yields. These were 10% ~ 15% and 15% ~ 20% improvements over the unrefined controls for glucose and xylose yield, respectively.

The highest glucose and xylose yields occurred in corn stover samples that were both deacetylated and mechanically refined. Under these conditions, both corn stover varieties achieved over 90% yields for both glucose and xylose.

In general, results from enzymatic hydrolysis at a 20% solids loading follow the same trend as was found in low-solids experiments. Applying either deacetylation or mechanical refining improved glucose yields by 10% ~ 15% and xylose yields by 15% ~ 20%. The glucose yields increased by 15% ~ 20% and xylose yields increased by 25% ~ 30% when deacetylated corn stover was pretreated and mechanically refined.

### High-solids enzymatic hydrolysis with unwashed solids

**Figure 3:**
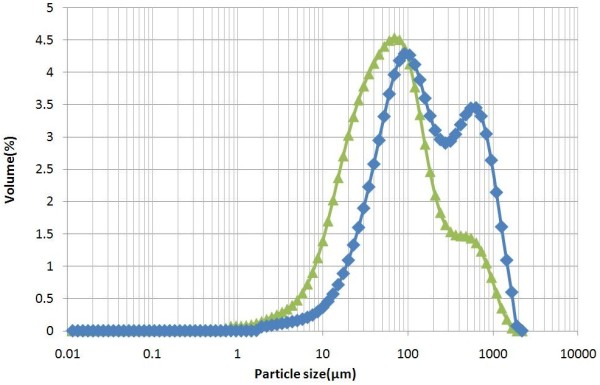
**Particle size distribution of control and mechanically refined corn stover; ‘blue diamond symbol’control; ‘green triangle symbol’PFI refined.**

Figure 3 shows the particle size distribution of pretreated corn stover before and after mechanical refining. While the unrefined corn stover has a volume mean particle size of 283 μm, after mechanical refining the mean particle size drops to 143 μm. While this reduction in particle size improves the enzymatic digestibility of the hydrolysate, it would also increase the cost of solid–liquid separation prior to enzymatic hydrolysis, as the cost of solid–liquid separation equipment increases for such small particles (<1 mm) [8]. Because of the high cost of solid–liquid separation at this stage, whole slurry high-solids enzymatic hydrolysis with unwashed solids was tested. This is also a direct comparison to the commercial-scale enzymatic hydrolysis process outlined in the NREL design report.

**Figure 4:**
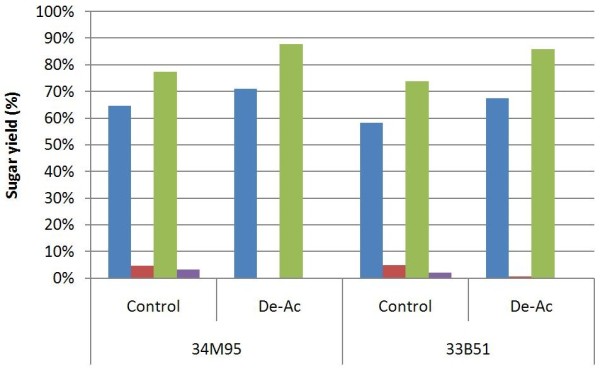
**High-solids enzymatic hydrolysis with whole slurry (non-washed solids); Enzyme is loaded as CT20; ‘blue square symbol’glucose yield, non-PFI refined; ‘red square symbol’xylose yield, non-PFI refined; ‘green square symbol’glucose yield, PFI refined; ‘purple square symbol’xylose yield, PFI refined.**

Enzymatic hydrolysis yields from experiments carried out at 25% total solids (18% insoluble solids) are shown in Figure 4. After enzymatic hydrolysis, the glucose concentrations were in the range of 120-140 g/L, while xylose concentrations were in the range of 50-70 g/L. The yield calculations are also based on the formula developed by Zhu et al. [25].

In all cases, very little monomeric xylose was produced during enzymatic hydrolysis. The high xylose concentration in the background liquor likely inhibited further hydrolysis of xylan in the solids or xylo-oligomers in the liquor. The high sugar product concentration can also stop the hydrolysis reaction thermodynamically by product inhibition.

The reduced xylose conversion also led to lower glucose yields when compared to results using washed PCS. The glucose yields for native control samples fall between 57% and 65%. Glucose yields for deacetylated samples range from 65% to 71%. Mechanical refining consistently improved glucose yields by more than 10% over unrefined samples. However, all yields are 5% ~ 10% lower than the yields for washed pretreated solids.

**Figure 5:**
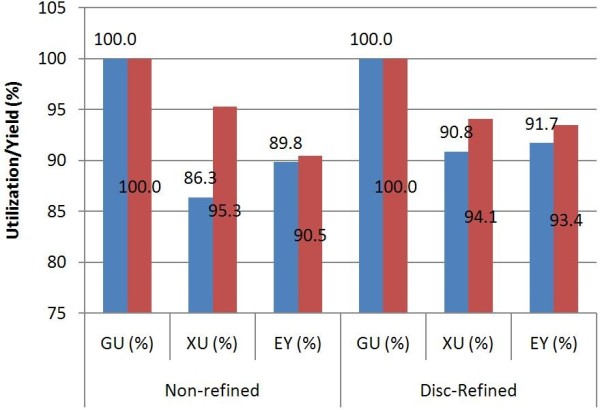
**Effect of deacetylation and mechanical refining on fermentation yield; ‘blue square symbol’control; ‘red square symbol’deacetylated; GU: glucose utilization; XU: xylose utilization; EY: ethanol yield.**

While improvements in monomeric sugar yields are promising, increased sugar concentrations alone will not improve process economics if the final product titer does not increase correspondingly due to hydrolysate toxicity or product inhibition. Therefore fermentability studies were executed to evaluate the viability of the overall process incorporating deacetylation and mechanical refining. Figure 5 shows the glucose and xylose utilization and ethanol yields after 30 h fermentation using whole slurry hydrolysates from Kramer 34 M95. After 30 h, 100% glucose utilization was achieved in all four cases (control, De-Ac, control refined, De-Ac and refined). Control samples realized a xylose utilization around 86% ~ 91%, while De-Ac samples utilized ~95% of available xylose. The overall ethanol yields ranged from 90% ~ 93% in all four cases. The highest ethanol concentration observed was 70 g/L for the deacetylated and mechanically refined PCS.

### Ethanol yield from one ton of biomass

Table 3 compares the amount of ethanol produced from 1 ton of dry Kramer 34 M95 corn stover reported in this study with the ethanol yield stated in the 2011 NREL design report. The theoretical maximum amount of ethanol produced from 34 M95 is 108 gal ethanol /ton of dry biomass. The highest-yielding process configuration identified in this study (washed, mechanically refined, deacetylated PCS; case 8) achieved an overall yield of 88.2 gal ethanol/ton of dry biomass, or roughly 81% of the theoretical yield. The unwashed case (case 4) combined with deacetylation and mechanical refining approached the same yield, achieving 82.2 gal ethanol/ton of dry biomass, 76% of the theoretical yield. The control case with unwashed PCS (case 1) resulted in an ethanol yield of only 63.9 gal ethanol/ton of dry biomass, equivalent to 59% of the theoretical conversion.

**Table 3:** Comparison of ethanol yield per dry ton of biomass for cases with and without washed solid process options, Ctrl = Control, DA = Deacetylated, MR = Mechanical Refining

	**Ethanol yield (gal/dry ton biomass)**							
	**No washing**				**W/ washing**			
	**1)Ctrl**	**2)DA**	**3)MR**	**4)DA + MR**	**5)Ctrl**	**6)DA**	**7)MR**	**8)DA + MR**
2011design	79.0							
34 M95	63.9	73.4	70.4	82.2	71.1	82.4	78.4	88.2

Ethanol yields in cases 4, 6, and 8 all exceed the 2011 design case, and case 7 nearly matches it. All these process scenarios include deacetylation and/or mechanical refining, offering an indication of the value of deacetylation and mechanical refining in a low-severity dilute acid pretreatment process. The hydrolysate wash stage has also proven to have a large role in maximizing ethanol yield. However, the overall cost of implementing these technologies on the commercial scale is unknown. In order for these scenarios to be practically included in the cellulosic ethanol design case, the increased ethanol yield must offset the additional capital and operational costs. This issue is addressed in our companion paper [21], where a techno-economic analysis is performed using the data developed in this study.

## Conclusions

Low-severity pretreatment at reduced temperatures and acid loadings is one way to mitigate these effects. However, low-severity pretreatment historically suffers from low monomeric xylose yields and cellulose digestibility. To solve these problems, deacetylation, mechanical disk refining, and solids washing were investigated and tested in numerous combinations using two varieties of corn stover.

Deacetylation is shown to increase xylose monomer yield during pretreatment but also to improve insoluble solids digestibility. Mechanical refining also has a positive impact on glucose and xylose yields during enzymatic hydrolysis. Combining both unit operations with low-severity pretreatment resulted in glucose yields of over 90% in low-solids enzymatic hydrolysis and in high-solids enzymatic hydrolysis using washed solids.

The washing of pretreated solids was also shown to be critical to the enzymatic conversion of residual xylan in the pretreated solids.

The reduction in acetic acid concentration from deacetylation in combination with lower pretreatment severity also improved the xylose utilization and ethanol yield in fermentation, ultimately achieving over 90% ethanol yield in less than 30 hours.

Deacetylation, mechanical refining, and solids washing has proven to be effective in improving ethanol yield. However, the commercial-scale viability of these additional unit operations needs to be evaluated to confirm that the resultant yield increases offset the increased capital and operational costs they present. In our companion paper, data provided by these experiments is used to perform a techno-economic analysis to evaluate the process economics, and the impact of these process modifications on the MESP is discussed [21].

## Methods

### Feedstock

In this study, two different varieties of corn stover feedstock were used. Those were from the Kramer farm in Wray, Colorado (Pioneer varieties 34 M95 and 33B51).

### Deacetylation

Deacetylation of the corn stover was performed by alkali extraction in a 200 L Recirculating Atmospheric Pressure Impregnation (RAPI) system. Approximately 10 kg of dry (~93 wt% solids), ½-inch (34 M95, 33B51) knife-milled corn stover was loaded into Hastelloy C-276 wire mesh (20 mesh screen) baskets and immersed in the recirculation bath containing 120 L of 0.4 wt% NaOH (0.1 M) solution at 70°C to 80°C for 3 hours. The initial pH was approximately 12. The solid-to-liquid ratio was 1:12. The recirculation helps to distribute alkali solution evenly throughout the basket and helps to provide efficient extraction of the corn stover feedstock. After extraction, the excess alkali solution was drained and a sample was retained for pH, acetate, and sugar concentration measurements. The alkali-extracted corn stover was washed with four separate washes (120 L) of warm water (~60°C) for 1 h, with the basket drained to ~15 wt% solids in between each wash, until the pH of the washed liquor measured ~8.0. The alkali-extracted and washed feedstock was used for dilute acid soaking as described below.

### Acid impregnation

Acid impregnation was carried out using the same 200-L recirculating (RAPI) system as described above. Approximately 120 L of warm (48°C to 50°C), 0.5 wt% H_2_SO_4_ was prepared beforehand in the recirculation tank. A Hastelloy C-276 wire mesh (20 mesh screen) basket was loaded with 50 kg of native or deacetylated and washed corn stover feedstock and lowered into the warm (48°-50°C) dilute acid bath for 3 hours with recirculation of the acid. Following acid impregnation, the feedstock in the basket was drained of excess acid to approximately 20% solids and loaded into the mold of a hydraulic dewatering press, where the acid impregnated feedstock was pressed to ~40% to 48% solids.

### Bench-scale pretreatments

The 4-L NREL Digester (steam explosion reactor) was used for all pretreatment experiments. The NREL Digester was pre-heated to pretreatment temperature and then was loaded with 500.0 g of acid-impregnated and pressed feedstock and quickly heated (~5 to 10 sec) via direct steam injection to reaction temperature. Pretreatments were carried out at the lower severity conditions of 150°C, 0.5 wt% H_2_SO_4_, and 20 min residence times. The initial solids concentration of the acid impregnated and dewatered corn stover is approximately 40%.

### Mechanical refining

For mechanically refined samples, the biomass slurry was processed in a PFI refiner and run for 4,000 revolutions. This process significantly reduces the particle size of the solids. Mechanical refining was carried out in collaboration with Dr. Sunkyu Park at North Carolina State University using their bench-scale PFI refining mill. The pretreated biomass slurries were diluted to 20 wt% consistency and refined for a total of 4,000 revolutions at room temperature. Around 40 g O.D. dry material were refined in a single batch. The solids were not washed before refining.

### Enzymatic hydrolysis

Novozymes (Frankinton, NC) cellulases, CTec2, and hemicellulases, HTec2, were used in the current study. The enzyme loadings were added at varied levels as described in the results and discussion section.

For washed solids enzymatic hydrolysis, the pretreated slurry was thoroughly washed with water in centrifuge bottles until xylose was <0.01 g/L (measured by YSI), usually 6 times.

Low-solids enzymatic hydrolysis experiments were set up in 125 mL Erlenmeyer flasks containing 50 mM citrate buffer, pH 4.8, enzyme, and 1.0% glucan loading (w/v). Digestion conditions were 150 rpm at 50°C.

High-solids enzymatic hydrolysis experiments were performed in 125 mL wide-mouth polypropylene bottles (Thermo Fisher Scientific, Inc., Waltham, MA) loaded with 60 g of washed slurry at 20% insoluble solids. Novozyme CTec2 is loaded at 20 mg protein per g of cellulose, while Novozyme HTec2 is loaded at 2 mg protein per g of cellulose. The roller bottle saccharification reactors employ gravitational tumbling as a mixing mechanism to homogenize solids by horizontally rotating the reaction vessels at 4 rpm on a 3-deck roller apparatus for mini bottles (Wheaton Industries Inc., Millville, NJ). The roller apparatus was placed in a general purpose incubator (Model 1545, VWR International, LLC, West Chester, PA) for temperature control at 48.5°C.

In whole slurry enzymatic hydrolysis, PCS was adjusted to a pH of 5.0 by adding small amounts of ammonium hydroxide and mixing well. No citrate buffer was added due to the inhibitory effect of citrate ions on *Zymomonas mobilis* growth and fermentation. Saccharification experiments were carried out in 125 mL wide-mouth polypropylene roller bottles with pH-adjusted slurry at 25% total solids. The other conditions are the same as for high-solids enzymatic hydrolysis using washed solids.

### Microorganism and revive/pre-seed culture

*Zymomonas mobilis* strain 8b was used in this evaluation. The strain was taken from cell stock stored at −70°C. The pre-seed medium consisted of 10 g/L yeast extract and 2 g/L potassium phosphate monobasic (1X RM), supplemented with 100 g/L glucose and 20 g/L xylose. The reviving culture was started by transferring 1 mL of *Z. mobilis* cell stock into 9 mL of pre-seed medium in a 15 mL tube. The culture was incubated at 33°C with no agitation. The culture was sampled at 8 hours for an optical density reading at 600 nm. The pre-seed culture was used to inoculate the batch seed fermenter with media composition of RM (1X), 150 g/L glucose; 20 g/L xylose; and 1 g/L sorbitol. pH and temperature were controlled at 5.8 by KOH (4 N) and at 33°C.

### Fermentation

Fermentation experiments to evaluate the neutralized saccharified whole slurry were performed in BioStat-Q Plus fermenters at a 300 mL working volume using r*Z.mobilis* strain 8b. Rich media consisting of 10 g/L yeast extract and 2 g/L KH_2_PO_4_ was added to enzymatically-hydrolyzed whole slurry. The fermenters were inoculated at an optical density (@ 600 nm) of approximately 1.0 absorbance units using a direct transfer procedure (10% v/v). The fermentation was conducted at a temperature of 33°C, a pH of 5.8 (controlled with 4 M KOH), and an agitation speed of 300 rpm. The fermentation was typically finished in 72 h. Ethanol yield calculations were based on initial glucose, xylose, and fructose concentrations and differences between initial and final ethanol concentrations.

### Chemical analysis and yield calculations

Pretreatment and enzymatic saccharification liquors and fermentation samples were analyzed using HPLC according to standard NREL laboratory analytical procedures (LAPs) [26]. Solid residues were also analyzed in accordance with the published NREL LAPs [26,27]. Sugar yields from high solids enzymatic hydrolysis were calculated using the equations developed by Zhu et al. [25].

In the liquor phase of pretreated slurry, acetate is present in two forms: 1) free acetate/acetic acid, released from the xylan; and 2) acetyl groups covalently bound to the dissolved xylan oligomers. Free acetate is measured by direct injection on a Shodex SP0810 acid column (Kawasaki, Japan), while total acetate is measured with the same column after a 4% acid hydrolysis of the filtered pretreatment liquid that contains all solubilized compounds. The 4% acid hydrolysis at 121°C for 1 h hydrolyzes the remaining acetyl groups covalently bound to the xylooligomers.

### Particle size analysis

The particle size of biomass samples was measured using laser diffraction on a Mastersizer 2000 with the Hydro 2000 G module (Malvern Instruments). The instrument measures particle sizes over the range from 0.02 to 2000 μm in a recirculating liquid suspension. For the analysis, 0.05 to 0.2 g of each cellulose sample was dispersed in water in a 15-mL centrifuge tube. Thereafter, individual dispersed samples were vortex mixed and transferred to the Hydro 2000 G module that contained 0.8 – 1.0 L of deionized water (*n*_r_ = 1.33 at 20°C), with a stirrer setting of 600 RPM and a pump setting of 1250 RPM. After 30 seconds delay, three, 15 second readings (30 seconds apart) of the circulating samples were acquired and averaged. The volume weighted mean value was used to represent the mean particle diameter (MPD). Each sample was run in triplicate and MPD is shown as the average of the triplicates.

## Abbreviations

MESP: Minimum ethanol selling price; NREL: National renewable energy laboratory; EIA: Energy information administration; TEA: Techno economic analysis; De-Ac: Deacetylation; PCS: Pretreated corn stover; CT10: Enzymatic hydrolysis with CTec2 10 mg protein /g of cellulose and HTec2 1 mg protein /g of cellulose loading; CT20: Enzymatic hydrolysis CTec2 20 mg protein /g of cellulose and HTec2 2 mg protein /g of cellulose loading; CT40: Enzymatic hydrolysis CTec2 40 mg protein /g of cellulose and HTec2 4 mg protein /g of cellulose loading; CT60: Enzymatic hydrolysis CTec2 60 mg protein /g of cellulose and HTec26 mg protein /g of cellulose loading; RAPI: Recirculating atmospheric pressure impregnation; MPD: Mean particle diameter.

## Competing interests

The authors declare that they have no competing interests.

## Authors’ contributions

XC designed and conducted the experimental work including deacetylation, pretreatment and enzymatic hydrolysis. He also analyzed the data and drafted the manuscript. LT helped design this work. JS co-conducted the experiments, reviewed results and finalized the manuscript. AM and HS did the fermentation work. SD helped design enzymatic hydrolysis and discussed part of the results. WW did the particle size measurement. SP generously helped provided the PFI equipment at NCSU and was involved in developing mechanical refining process. MH originated and managed the first biomass wet and dry milling research program at NREL which provided the foundation and guidance for this work. MT led and coordinated the overall project. MT also conducted the pretreatment experiments along with XC. All authors have read and approved the final manuscript.

## Authors’ information

Dr. Xiaowen Chen received his master’s and Ph.D’s degree in chemical engineering from University of Maine. He is now a chemical engineer at the National Renewable Energy Lab. His research interest is in process development and biochemical engineering in cellulosic ethanol.

## References

[B1] U.S. Energy Information Administration Annual Energy Outlook 2011 with Projections to 2035 2011 DOE/EIA-0383

[B2] Foust TD Wooley R Sheehan J Wallace R Ibsen K Dayton D Himmel M Ashworth J McCormick R Melendez M A National Laboratory Market and Technology Assessment of the 30x30 Scenario. DOE Report, NREL/TP-510-40942 2007 Golden, CO: National Renewable Energy Laboratory

[B3] Humbird D Davis R Tao L Kinchin C Hsu D Aden A Schoen P Lukas J Olthof B Worley M Process Design and Economics for Biochemical Conversion of Lignocellulosic Biomass to Ethanol: Dilute-Acid Pretreatment and Enzymatic Hydrolysis of Corn Stover. Technical Report, NREL/TP-5100-47764 2011 Golden, CO: National Renewable Energy Laboratory

[B4] Nagle N Kuhn E Elander R Parametric study using the modified continuous horizontal pretreatment reactor using corn stover to determine operating conditions that can achieve yields of ≥ 85% of monomeric xylose 2010 National Renewable Energy Lab: In preparation

[B5] Franden MA Pienkos PT Zhang M Development of a high-throughput method to evaluate the impact of inhibitory compounds from lignocellulosic hydrolysates on the growth of Zymomonas mobilis J of Biotechno 2009 144 259 267 10.1016/j.jbiotec.2009.08.006 19683550

[B6] Chen X Shekiro J Elander R Tucker MP Improved Xylan Hydrolysis of Corn Stover by Deacetylation with High Solids Dilute Acid Pretreatment Ind Eng Chem Res 2012 51 6

[B7] Chen X Shekiro J Franden M Wang W Zhang M Kuhn E Johnson D Tucker M The impacts of deacetylation prior to dilute acid pretreatment on the bioethanol process Biotechnology for Biofuels 2012 5 8 10.1186/1754-6834-5-8 22369467 PMC3309953

[B8] Aden A Ruth M Ibsen K Jechura J Neeves K Sheehan J Wallace B Montague L Slayton A Lukas J Lignocellulosic Biomass to Ethanol Process Design and Economics Utilizing Co-Current Dilute Acid Prehydrolysis and Enzymatic Hydrolysis for Corn Stover Technical Report, NREL/TP-510-32438 2002 Golden, CO: National Renewable Energy Laboratory

[B9] Zhang J Song H Lin L Zhuang J Pang C Liu S Microfibrillated cellulose from bamboo pulp and its properties Biomass Bioenergy 2012 39 78 83

[B10] Zhu W Zhu JY Gleisner R Pan XJ On energy consumption for size-reduction and yields form subsequent enzymatic saccharification of pretreated lodgepole pine Bioresour Technol 2010 101 2782 2792 10.1016/j.biortech.2009.10.076 20006490

[B11] Koo B-W Treasure TH Jameel H Philips RB Chang H-m Park S Reduction of Enzyme Dosage by Oxygen Delignification and Mechanical Refining for Enzymatic Hydrolysis of Green Liquor-Pretreated Hardwood Appl Biochem Biotechnol 2011 165 832 844 10.1007/s12010-011-9301-4 21647684

[B12] Kerekes RJ CHARACTERIZING REFINING ACTION IN PFI MILLS 2002 TAPPI Paper Summit

[B13] Wang X Maloney TC Paulapuro H Internal fibrillation in never-dried and once-dried chemical pulps Book Internal fibrillation in never-dried and once-dried chemical pulps 2002 469 473

[B14] Laine C Wang. X, Tenkanen M, Varhimo A: Changes in the fiber wall during refining of bleached pine kraft pulp Holzforschung 2004 58 233 240

[B15] Hui L Liu Z Ni Y Characterization of high-yield pulp (HYP) by the solute exclusion Bioresour Technol 2009 100 6630 6634 10.1016/j.biortech.2009.07.055 19692226

[B16] Huang R Su R Qi W He Z Understanding the key factors for enzymatic conversion of pretreated lignocellulose by partial least square analysis Biotechnol Prog 2010 26 384 392 19938060 10.1002/btpr.324

[B17] Chandra RP Bura R Mabee WE Berlin A Pan X Saddler JN Substrate pretreatment: the key to effective enzymatic hydrolysis of lignocellulosics? Biofuels 2007 108 67 93 10.1007/10_2007_064 17530205

[B18] Grethlein H The effect of pore size distribution on the rate of enzymatic hydrolysis of cellulosic substrates Biotechnology 1985 3 155 160

[B19] Grethlein HE Converse AO Commen aspects of acid prehydrolysis and steam explosion for pretreating wood Biores Technol 1991 36 77 82 10.1016/0960-8524(91)90101-O

[B20] Mooney CA Mansfield SD Touhy MG Saddler JN The effect of initial pore volume and lignin content on the enzymatic hydrolysis of softwoods Bioresour Technol 1998 64 113 119 10.1016/S0960-8524(97)00181-8

[B21] Tao L Chen X Kuhn E Tucker M Aden A Elander R Improved ethanol yield and reduced MESP by modifying low severity dilute acid pretreatment with deacetylation and mechanical refining: 2) Techno-economic analysis In Press 10.1186/1754-6834-5-69 PMC3478232 22967479

[B22] Zhu YM Lee YY Elander RT Dilute-Acid Pretreatment of Corn Stover Using a High-Solids Percolation Reactor Appl Biochem Biotechnol 2004 117 103 114 10.1385/ABAB:117:2:103 15159554

[B23] Tucker MP Kim KH Newman MM Nguyen QA Effects of Temperature and Moisture on Dilute-Acid Steam Explosion Pretreatment of Corn Stover and Cellulase Enzyme Digestibility Appl Biochem Biotechnol 2003 105-108 165 177 12721483 10.1385/abab:105:1-3:165

[B24] Humbird D Mohagheghi A Dowe N Schell DJ Economic Impact of Total Solids Loading on Enzymatic Hydrolysis of Dilute Acid Pretreated Corn Stover Biotechnol Prog 2010 26 1245 1251 10.1002/btpr.441 20945482

[B25] Zhu YM Malten M Torry-Smith M McMillan JD Stickel JJ Calculating Sugar Yields in High Solids Hydrolysis of Biomass Bioresour Technol 2011 102 2897 2903 10.1016/j.biortech.2010.10.134 21109427

[B26] Sluiter JB Ruiz RO Scarlata CJ Sluiter AD Templeton DW Compositional Analysis of Lignocellulosic Feedstocks. 1. Review and Description of Methods J Agric Food Chem 2010 58 9043 9053 10.1021/jf1008023 PMC2923870 20669951

[B27] Templeton DW Scarlata CJ Sluiter JB Wolfrum EJ Compositional Analysis of Lignocellulosic Feedstocks. 2. Method Uncertainties J Agr Food Chem 2010 58 9054 9062 10.1021/jf100807b PMC2923869 20669952

